# The integrated care pathway for post stroke patients (iCaPPS): a shared care approach between stakeholders in areas with limited access to specialist stroke care services

**DOI:** 10.1186/s12913-016-1963-8

**Published:** 2017-01-13

**Authors:** Aznida Firzah Abdul Aziz, Nor Azlin Mohd Nordin, Mohd Fairuz Ali, Noor Azah Abd Aziz, Saperi Sulong, Syed Mohamed Aljunid

**Affiliations:** 1 Department of Family Medicine, 14th Floor, Preclinical Block, Faculty of Medicine, Universiti Kebangsaan Malaysia, Jalan Yaacob Latiff, Bandar Tun Razak, Cheras, 56000 Kuala Lumpur Malaysia; 2 School of Rehabilitation Sciences, Faculty of Health Sciences, University Kebangsaan Malaysia, Kuala Lumpur, Malaysia; 3 Department of Health Information, Faculty of Medicine, Universiti Kebangsaan Malaysia Medical Centre, Cheras, Kuala Lumpur Malaysia; 4 International Centre for Casemix and Clinical Coding, Faculty of Medicine, Universiti Kebangsaan Malaysia Medical Centre, Cheras, Kuala Lumpur Malaysia; 5 Department of Health Policy and Management, Faculty of Public Health, Kuwait University, Kuwait, Kuwait

**Keywords:** Secondary prevention adherence stroke, Community based, Post stroke complications, Developing countries, Health services, Health care, Prevention of post stroke sequelae, Rehabilitation

## Abstract

**Background:**

Lack of intersectoral collaboration within public health sectors compound efforts to promote effective multidisciplinary post stroke care after discharge following acute phase. A coordinated, primary care-led care pathway to manage post stroke patients residing at home in the community was designed by an expert panel of specialist stroke care providers to help overcome fragmented post stroke care in areas where access is limited or lacking.

**Methods:**

Expert panel discussions comprising Family Medicine Specialists, Neurologists, Rehabilitation Physicians and Therapists, and Nurse Managers from Ministry of Health and acadaemia were conducted. In Phase One, experts chartered current care processes in public healthcare facilities, from acute stroke till discharge and also patients who presented late with stroke symptoms to public primary care health centres. In Phase Two, modified Delphi technique was employed to obtain consensus on recommendations, based on current evidence and best care practices. Care algorithms were designed around existing work schedules at public health centres.

**Results:**

Indication for patients eligible for monitoring by primary care at public health centres were identified. Gaps in transfer of care occurred either at post discharge from acute care or primary care patients diagnosed at or beyond subacute phase at health centres. Essential information required during transfer of care from tertiary care to primary care providers was identified. Care algorithms including appropriate tools were summarised to guide primary care teams to identify patients requiring further multidisciplinary interventions. Shared care approaches with Specialist Stroke care team were outlined. Components of the iCaPPS were developed simultaneously: (i) iCaPPS-Rehab© for rehabilitation of stroke patients at community level (ii) iCaPPS-Swallow© guided the primary care team to screen and manage stroke related swallowing problems.

**Conclusion:**

Coordinated post stroke care monitoring service for patients at community level is achievable using the iCaPPS and its components as a guide. The iCaPPS may be used for post stroke care monitoring of patients in similar fragmented healthcare delivery systems or areas with limited access to specialist stroke care services.

**Trial registration:**

No.: ACTRN12616001322426 (Registration Date: 21st September 2016).

## Background

Stroke is a major global public health problem in terms of mortality and disability, and its incidence is expected to increase over the next 20 years as the world population ages [1, 2]. Advancements in medical technologies have improved the mortality rates among stroke patients, resulting in one third of afflicted stroke patients worldwide surviving with permanent disabilities in their advancing years. Although stroke is an acute affliction, its long-term complications and disabilities require long-term dependency on the public health care system. Post stroke care delivery requires the coordination of multidisciplinary service provision; intersectoral collaboration within the public health care delivery system is vital to ensure that patients continue to receive optimal post stroke care once they are discharged from hospital following acute stroke. In most public health systems around the world, fragmentation and poor coordination of care have been the greatest obstacles in ensuring adequate post stroke care and rehabilitation [3, 4].

Developing countries face added challenges in terms of access to specialized stroke care services and the lack of adequately trained staff members to provide continuity of stroke rehabilitation once patients are back in the community [1, 5–8]. Malaysia, in its bid to achieve the status of a developed nation, has a fairly well-established public health system to cater to its expanding population [9]. However, a review of the 10th Malaysian Plan revealed problems associated with access to and inequity in specialized care (such as stroke care services), and consolidation of the current health care service has been identified as a solution [10, 11].

The lack of data on the national prevalence and long-term outcomes of stroke [12] patients in this country calls for better coordination among the providers of stroke care. To date, data on in-patient admissions for stroke are only available from the northern parts of Malaysia – mainly from Penang and Terengganu [13, 14]. As post stroke care is multidisciplinary, there is a need to optimize the current health care delivery system to benefit stroke survivors and their caregivers. The public primary health care facilities in this country are well equipped to provide chronic long-term care. Trained family physicians or family medicine specialists (FMS) at most of the 220 public health centers across the country have led the primary care team to provide quality primary health care services at various health centers. These health centers also provide physiotherapy and/or occupational therapy, medical laboratory services, as well as on-site dispensing pharmacy services. These health centers provide primary care services to the surrounding communities in areas with populations ranging from 10,000–30,000 people. The current literature from developed countries mainly addresses acute in-patient care and post stroke care provision in communities that are supported by community-based rehabilitation centers and social support services [4, 15–18]. These facilities are primarily scarce or absent in most developing countries across the globe, and they largely depend on primary care teams to provide adequate, accessible health care services to the population.

Integrated care pathways (ICP) are structured multidisciplinary care plans that map out the expected care for patients of a clinical condition [19]. It describes how care should be structured and coordinated, outlining standards during the course of care based on evidence and best practices. Historically, ICPs originated in the United States of America and the United Kingdom in the 1990s and they are widely used in developed countries. ICPs were initially designed for the in-patient care of illnesses and they are usually unique to the institution or hospital in which they were developed. All variations from the pathway are documented, and the reasons for the variations have been analyzed. Solutions are developed to address the causes of potentially avoidable variations, and the pathway is revised to incorporate these improvements. In Malaysia, the conventional care model that is currently in place lacks direction in terms of its ability to organize rehabilitation for post stroke patients at the community level, as well as to screen for stroke-related complications (refer to Table 1). Hence, we believe that having an Integrated Care Pathway for Post Stroke Patients (iCaPPS) residing at home in the community is a positive undertaking among the specialist stroke care providers, and that it should be implemented in collaboration with the primary care team. It is hoped that the iCaPPS will also serve as a catalyst to improve the quality of stroke care delivery for developing countries with similarly challenged public health systems.

**Table 1:** Post Stroke Care At Public Primary Care Healthcentres (Reproduced with permission from Aznida FAA, 2015)

Assessment/Treatment	Monitoring/screening procedure	Conventional Care
Stroke Risk factor(s) monitoring or NCD monitoring	Vital signs (blood pressure, pulse rate)	+
	Fasting blood sugar	+
	HbA_1c_	+
	Fasting blood lipids	+
	Renal function	+
	eGFR	±
Screening for stroke-related complications	Functional status	-
	IADL	-
	Depression	-
	Cognitive assessment	-
Rehabilitation intervention/assessment of progress	Physiotherapy	±
	Occupational therapy	±
	Speech/language therapy	-

*eGFR* estimated (calculated) Glomerular Filtration Rate

+ done on a regular basis, ± occasionally done in some health centres

- not done on a regular basis

## Methods

The pathway design involved an expert panel discussion to provide input on the care of stroke patients from the acute stage to long-term care, from the stroke care providers’ perspective. The iCaPPS design mainly focused on the stroke care providers’ perspective, and used the input from the stroke patients and their caregivers’ perspective from an earlier study as a basis for discussion by the expert panel [20].

This was a two-phase process: (i) Phase 1 aimed to outline the current care process for the management of patients presenting with stroke to the public health care facilities in Malaysia. This is because the current existing clinical practice guidelines did not address issues of transfer of care beyond acute stroke care, long-term care, or further rehabilitation of stroke survivors at the community level. (ii) In Phase 2, the panel was asked for their opinion on the management of stroke patients at the community level, so as to include recommendations based on the current evidence and to determine the panel’s suggestions for best care practices that would be suited for the local public health care system.

### Expert panel selection

The experts were recruited to represent the multidisciplinary stroke health care providers at the Ministry of Health, as well as to represent academicians based at teaching hospitals. Directors and heads of specialties and related professional associations were consulted to nominate the most appropriate person to be invited for the panel discussion. All experts were clinicians who were actively involved in providing stroke care in all public as well as university healthcare facilities. The number of health professionals involved in provision of stroke care in this country is extremely limited, hence the necessity to identify the relevant experts. Due to heterogeneity of stroke care provision in the country at the time this study was conducted, and based on the objective of the study to provide post stroke care monitoring for patients in the community, front liners to stroke care provision were prioritized and invited to join the panel discussion. In this case, Family Medicine Specialists, community based therapists and nurses based at primary care health centres were identified by respective professional bodies before invitations were issued to join the panel. Specialist stroke care providers in the private sector were not considered, as the aim of this initiative was to improve the current post stroke care delivery at public health care facilities. Each of the expert panel members had a minimum of 5 years’ working experience as a specialist in his or her respective discipline. The experts were grouped, as listed below, according to their areas of expertise.

- **Expert Panel 1**: Consultant neurologists with special interest in stroke care (3), FMS, and the current president of the Malaysian Family Medicine Specialists Association (FMSA) (5). Altogether, six neurologists were invited to attend the meeting; three declined due to clashing prior commitments. The five family medicine consultants that were selected were those with fellowship training in chronic non-communicable disease (NCD) care, geriatric care, community mental health, and stroke rehabilitation.
- **Expert Panel 2**: Family medicine consultants (2 individuals), rehabilitation physicians (2 individuals), psychiatrists (2 individuals), physiotherapists (2 individuals), occupational therapists (2 individuals), speech and language pathologists (4 individuals), and nurses (3 individuals).

The family medicine consultants in this group had fellowship training in community stroke rehabilitation and community geriatric care. The psychiatrists were trained in psychogeriatrics and community psychiatry. All of the experts represented resource persons in Malaysia, combining experts from both the Ministry of Health and the Ministry of Higher Education, who were clinicians and actively working in providing specialist stroke care services for the country at public healthcare facilities.

### Phase 1

Face-to-face expert panel discussions for both groups were held on April 9, 2012 and April 16, 2012, respectively. Both sessions took place at the United Nations University International Institute for Global Health, Kuala Lumpur. Experts were given an overall briefing on the objectives of the meeting and details on the expected outcomes – i.e., developing the Integrated Care Pathway for Post Stroke (iCaPPS) for patients residing at home in the community. The researcher briefed the experts on the findings of earlier studies performed at local public primary care facilities and on self-reported practices among FMS in managing stroke patients [21, 22]. The experts outlined the usual care processes that are currently in practice in most public health care facilities across the country, describing the process of care from the acute stage until discharge from a tertiary center; they also outlined the subsequent follow-up process that continues in the outpatient setting of the multidisciplinary services required by the patients. The panel further identified a group of patients who present to primary care centers late after a stroke, without any prior acute treatment at a tertiary center. This phase provided the expert panel with a framework through which to identify the gaps in care delivery, which may be improved upon. The panel discussed the post stroke care plan using the framework for rehabilitation of stroke patients as a basis i.e. (1) to manage the stroke risk factors, (2) to prevent complications related to stroke and/or non-stroke related, (3) reintegrate the survivors back into the community. The discussion allowed panel members to be orientated to the various members of the multidisciplinary team, which manage the stroke patient once the patient, is discharged after the acute stroke episode. All decisions made by the panel members during the face-to-face discussions were finalized only after a consensus decision was reached.

The sessions were taped on audio recorders and discussions on care algorithms were illustrated using flipcharts as the meeting progressed. The audio taped discussions were verified and compared with the illustrated charts by the researcher and another co-author (NAMN), to determine completeness.

### Phase 2

**Fig. 1:**
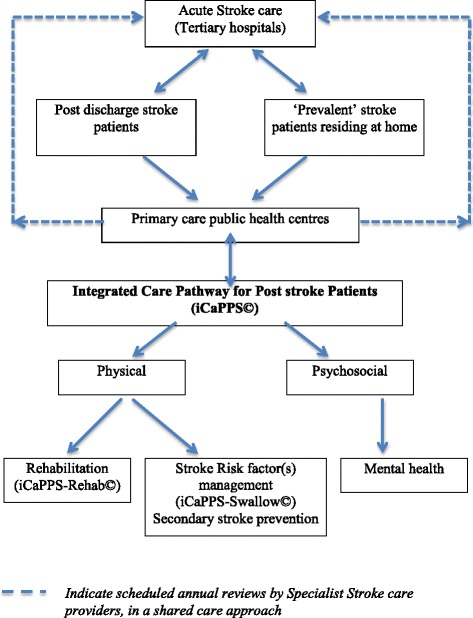
Post stroke care model for patients residing at home in the community (Reproduced with permission from Aznida FAA, 2015)

In this phase, the care algorithms that were mapped out in Phase 1 were then modeled to address both the physical and psychosocial components of post stroke management at the community level (Fig. 1). The current post stroke care provision at these public primary care health centers primarily focuses on the management of stroke risk factors, with rehabilitation aspects mainly not coordinated or made known to the receiving primary care team due to poor transfer of care from tertiary to primary care health teams.

A modified Delphi technique was employed to obtain members’ detailed comments and feedback on items that represented additional recommendations to provide holistic approach to the management of post stroke patients at community level. The recommendations that were suggested were based on the current medical literature and different health care delivery systems or local protocols for managing stroke. Individually addressed electronic mails were sent to the same members of the panel, to obtain a consensus regarding the adoption of the recommended surveillance, screening tools and best care practices under the prevailing local health care delivery system. This method was agreed by members of the same expert panel as the most convenient method to reach a consensus for issues, which may have risen after the face-to-face discussion. The majority of expert panel members were mostly working across the country and attempts to schedule further meetings were unsuccessful. Hence, as an alternative option, the members agreed to be contacted via email, and were asked to identify their preferred emails which would facilitate prompt responses. The members were asked to provide their opinions on the highlighted issues, as well as their reasons behind these opinions, to the researcher; they were not aware of each other’s opinions. Expert opinion on issues such as: period of transfer of care between tertiary to primary care was obtained after two rounds of discussion among members, for rehabilitation intervention for post stroke patients (i.e. leisure intervention) also required two rounds of discussion and screening for swallowing disorders took altogether four rounds of discussion before a consensus agreement was reached. The findings of the consensus was finalised and the pathway and accompanying documents were compiled and distributed for approval.

The pathway developed from this project represents the first detailed guide that will steer a shared care approach between primary care and specialist stroke care providers for patients residing at home in the community.

## Results

### Profile of post stroke patients who can be managed at primary care public health centers

Expert panel 1 members identified the profile or criteria of patients who could be managed by the primary care team at public health centers. In view of the lack of standardized discharge protocols in various parts of the country, the group decided to commence discussion by working out the pre-discharge pathway to gain some insight and to identify patients who would benefit from long-term monitoring at primary care facilities, based on best practice criteria and their working experience at their institutions. The panel members first shared the protocol used to discharge post stroke patients after the acute episode, using what was the current standard operating procedure at their place of practice. A consensus decision was then reached on the criteria of patients who could be managed at primary healthcare facilities in a shared care approach and those who needed to be on long term follow up at the Specialist/tertiary outpatient clinic. Patients who met the following prerequisites could be followed up at public primary care health centers: *patients aged 40 years or older at the time of acute stroke; those who do not have concurrent cardiovascular disease; those with normal kidney function; those with coronary artery stenosis with a lumen patency greater than 50%; and those with stroke risk factors or comorbid conditions that are well controlled.* The panel unanimously agreed that younger patients i.e. aged 40 years and below, should be followed up at a tertiary centre with Specialist Stroke Care services as opposed to primary care facilities. Patients in these age groups usually require other specialist services, which are, which are mostly based at tertiary hospitals e.g. Cardiology or Endocrinology services. The criteria were applicable for all stroke types, and recommended to those who had access to the public primary healthcare centres across the country. The shared care approach allows for continued review of the patient between the specialist stroke care providers and the primary care team. It also facilitates long-term outcome assessment of the stroke patient once he or she leaves the tertiary/secondary hospitals, marking completion of the stroke cohort data capture for the National Neurology Registry, with aim to decentralize the long term care to the primary care team. Functional ability or transfer ability was not part of the criteria for the patients to be followed up at the primary care facilities, as this was the only available option for the stroke survivors and their caregivers to continue to get treatment once they are discharged. As opposed to health systems in developed countries, where assessments and support is made available for patients in the comfort of their own homes, the current public healthcare delivery in this country and most developing countries in this region, for stroke is lacking, and post stroke survivors have to depend on their caregivers to access medical care at the nearest public primary care health centres. Family Medicine Specialists lead a team of Medical & Health officers and supporting staff at the primary care public health centres. Services available at these health centres include an on-site dispensing pharmacy, diagnostic laboratory services, and emergency room facilities; Physiotherapist and/or Occupational therapist, visiting dietetic service complete the team at the health centres complex. Some of these health centres also have an attached oral health service. These health centres usually provide heavily subsidized healthcare services to surrounding communities with 20–30,000 people.

The principal discharging team, which consists of neurologists or physicians and rehabilitation physicians based at the tertiary or secondary hospital where the patient was treated during the acute stroke period, determines the best period during which transfer of care takes place. This is done once the identification and screening (i.e. echocardiography, carotid vessels assessment, renal function, dyslipidemia, and glycemic control assessments) of stroke risk factor(s) have been organized or completed.

In terms of rehabilitation, this should be organized *prior to discharge* and continued either at a hospital-based outpatient service or at community-based rehabilitation facilities. Coordination of rehabilitation is challenging as Stroke Units are not readily available at all tertiary hospitals in the country, and take on many different forms and definitions. Inadvertently, should the patient develop or is detected to have any of the aforementioned complications while undergoing treatment at a primary care center, he or she is to be referred to the nearest tertiary/secondary referral center for further management. In most cases, the shared care approach for chronic NCD is applied.

**Fig. 2:**
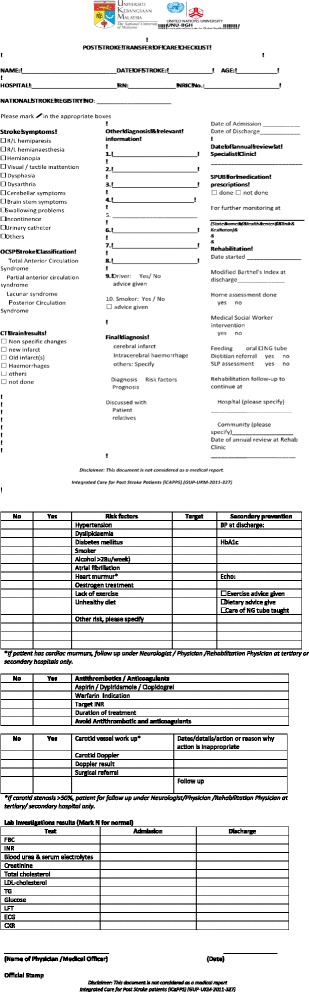
Post Stroke Transfer of Care Checklist (Reproduced with permission from Aznida FAA, 2015)

The panel listed all this information, which was highlighted as necessary; these details should be conveyed to the primary care center during the transfer process. The essential information is summarized in Fig. 2.

### Stroke risk factor monitoring

In this section, the panel outlined treatment targets for blood pressure, lipids [23] and glycemic control [24], which would follow current local clinical practice guidelines (CPG).

### Screening for stroke complications: The iCaPPS-Swallow© and the iCaPPS-Rehab© components

**Fig. 3:**
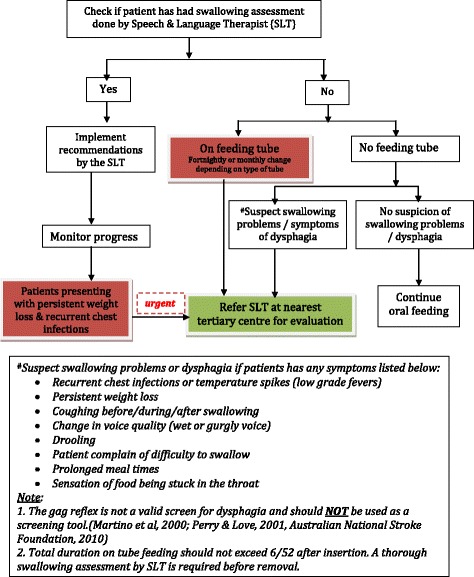
Management of Post stroke patients with possible swallowing problems at community level (iCAPPS-Swallow) (Reproduced with permission from Aznida FAA, 2015)

The panel highlighted five issues that require regular monitoring and surveillance during each follow-up visit; these include type of feeding, bowel habits, depression, dementia, and functional ability. In terms of problems with feeding, the greatest concern was the presence of a swallowing disability resulting after a stroke. The main issue associated with this was to decide when the nasogastric tube should be safely removed to resume oral feeding, especially among those who were discharged home with tube feeding. The appropriate assessments that should be performed at the community level to determine the safe and timely removal of the nasogastric tube are further outlined in the care algorithm for the detection of swallowing disorders. This algorithm also highlights the role of speech language therapists (SLTs) in assessing swallowing disorders. This topic led many to arrive at a consensus regarding issues pertaining to a lack of physician awareness on the roles of an SLT, as well as to access to services within the health care system, and the lack of trained personnel to safely conduct screening tests (which may cause harm to patients), which represent some of the issues addressed by the panel. Please refer to Fig. 3 for further details on the iCaPPS-Swallow©.

**Fig. 4:**
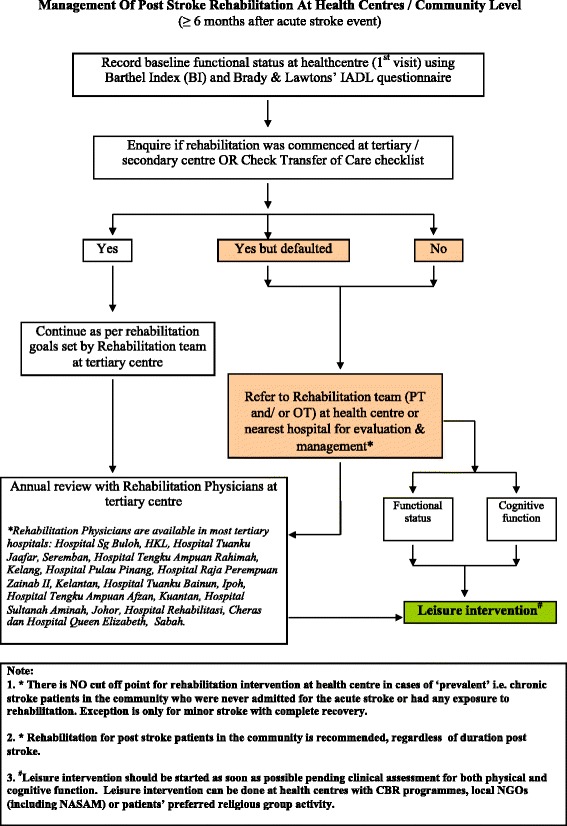
Rehabilitation of Post Stroke Patients at Community Level (iCaPPS-Rehab) (Reproduced with permission from Aznida FAA, 2015)

The iCaPPS-Rehab© addresses the management of functional status – i.e., the rehabilitation of post stroke patients. This algorithm is catered to those who were either initiated with rehabilitation (i.e., either physiotherapy and/or occupational therapy) while receiving treatment at hospitals during the acute stage, those who have defaulted rehabilitation for whatever reason, and those who have not had any rehabilitation since diagnosis. The latter group is usually comprised of patients who present to primary care centers at the later stages of stroke or with related complications, or among those described as having ‘prevalent’ stroke by Hare [25]. The main issue highlighted in this portion of the pathway is the importance of rehabilitation, regardless of the stage of recovery or the duration/time lapsed after the stroke, apart from those with minor stroke with complete recovery. The iCaPPS-Rehab© provides a safety net mechanism to ensure that rehabilitation is continued or initiated by the primary care team once the patients avail treatment at the public primary care health centers. Leisure intervention is also recommended in this pathway, advocating for use of available resources within the community. Please refer to Fig. 4.

### Caregiver screening

The panel agreed that caregiver screening was an important issue that should be addressed by the primary care team, but inquiries to determine caregiver strain should be performed once good rapport has been established with the patient and caregiver (i.e., from visit two onwards).

### The iCaPPS

Overall, the main iCaPPS addressed ten care issues that take place in a primary care setting once the patient is discharged from hospital. Among the areas covered are assessment by a FMS or medical officer, the monitoring of stroke risk factors, mood, cognition, medical investigations, nursing, the evaluation of swallowing disorders (i.e., to be completed by speech and language therapists, occupational therapists, or medical officers, whoever is available), physiotherapy, occupational therapy, and the list of medications used. The items to be addressed during each visit to the primary care or primary care public health centers and treatment targets are summarized in Table 2. In the table, there is a column to record variations in practice – i.e., of the items not covered during the visits and the reasons for non-adherence to the protocol. This portion was an addition to the iCaPPS document to facilitate further improvements and feedback. Table 2 summarizes the iCaPPS treatment targets and screening guidelines for post stroke patients residing at home in the community.

**Table 2:** Summary Of Expected Outcomes For the Integrated Care Pathway for Post Stroke patients (iCaPPS) residing at home in the community (Reproduced with permission from Aznida FAA, 2015)

	Care Plan	Items	Treatment Targets/Screening schedules^a^
Biological	Stroke risk factor(s) management	Hypertension	Lifestyle changes if BP between 130-139 mmHg systolic and/or 80–89 mmHg diastolic. Treat medically if BP >140/90 mmHg Target BP for diabetics is ≤ 130/80 mmHg Hypertension should be treated in the very elderly (age > 70 years) to reduce risk of stroke.
		Dyslipidaemia	For patients with ischaemic stroke: High risk group: keep LDL-cholesterol < 2.6 mmol/l or 2.0 mmol/L (considered in category of CVD or CHD risk equivalents) For patients with haemorrhagic stroke, the evidence is inconclusive.
		Diabetes mellitus	Tight glycaemic control is advised: HbA_1c_ <6.5% Fasting blood sugar 4.4–6.1 mmol/L
		Renal impairment/Chronic Kidney Disease	eGFR should be monitored at least annually, as in monitoring for patients with diabetes ellitus
	Rehabilitation	Physiotherapy	Please check if patient has been referred for rehabilitation prior to discharge from hospital. If patient has been referred, check compliance and ensure or organise annual review by Rehabilitation Physician. Goals for rehabilitation are based on discussion with patient and/or caregiver and Rehabilitation team. Refer to iCaPPS-Rehabilitation^a^ algorithm for further details.
		Occupational Therapy	
		Speech & Language Therapy	For patients on oral feeding, please check if screening for dysphagia was done during admission for acute stroke (includes evaluation by SLP). If not done, please check for symptoms of dysphagia at initial and subsequent visits. If patient is on nasogastric tube feeding at initial consultation, please check for duration and long-term feeding plans. Refer to iCaPPS-swallow^*^ for further details.
Psycho-social	Mental Health assessment	Depression	Screen patient for depression at initial and subsequent visits, using the TQWHQ. If positive, please proceed to use PHQ9 to determine level of intervention.
		Dementia	Screen with ECAQ for patients ≥ 60 years old. If score is <5, proceed with M- MMSE testing. M- MMSE for patients < 60 years old. If Score ≤ 17, to refer to Psycho-geriatrician for further evaluation and intervention.

^a^Based on recommendations by iCaPPS Expert Panel, Clinical Practice Guidelines of the Malaysian Academy of Medicine and Ministry of Health

*BP* blood pressure, *eGFR* calculated Glomerular Filtration Rate, *SLP* Speech and Language Pathologists or Therapists, *TQWHQ* Two Question with Help Questionnaire, *PHQ9* Patient Health Questionnaire (nine items). *M-MMSE* Mini Mental State Examination (Malay version)

## Discussion

In recent years, there has been a move to actually extend care pathways to cover long-term care and rehabilitation facilities (i.e., nursing homes and home health care), although literature in these areas is scarce [26, 27]. ICPs define the expected course of events in the care of a patient with a particular condition within a set timescale. Specific goals and expected progress at specific timelines are outlined together with appropriate investigations and treatment. The use of ICPs facilitates the introduction of guidelines, and promotes the continuous evaluation of clinical practices and workflow processes. Improvements are achieved by frequently revising the pathways to reflect current local best practices. ICPs differ from CPG, protocols, and algorithms, as a multidisciplinary care team uses them and they focus on the quality and coordination of care.

In the provision of stroke care, ICPs have been widely implemented in the management of acute stroke [28, 29]. However, the coordination of post stroke care beyond the hospital setting has been largely disjointed and inconsistently delivered [3, 4]. In Malaysia, the delivery of care beyond the acute phase has been challenging, as coordination of care between the multidisciplinary teams involved in post stroke rehabilitation is lacking. The lack of a centralized tracking mechanism may have detrimental effects on care, resulting from inconsistencies in the sharing of medical information during transfer of care between health care facilities [3]. Information on the long-term outcomes of stroke patients in this country are lacking [13] and, to a certain extent, the evaluation of the effectiveness of current service provisions is unknown.

In line with the National Strategic Plan for the management of Non-Communicable Diseases (NSPNCD) [10] there is now a greater emphasis on optimizing the provision of health management across stakeholders, including the shared management of these conditions between hospital care and primary health care specialists. The NSPNCD acknowledges that within the public health sectors, care delivery becomes disjointed when it involves intersectoral or transdisciplinary collaboration. Hence, the 9th Malaysian Plan (Health section) (2006–2010) [30] identifies the need to consolidate the current services to achieve better health for all as a priority. Intensifying resources and services by reinforcing multidisciplinary care for post stroke patients should be the focus.

The representation of a multidisciplinary panel of experts from both the Ministry of Health and the Ministry of Higher Education in the development of the iCaPPS will attempt to overcome the shortcomings of the disparity and variances in service provision between the two health care providers in this country. Evidence-based and best practices used in the delivery of care within the existing public health service system will enable realistic and workable adjustments to enhance the current health care delivery system for post stroke patients. The iCaPPS, to our knowledge, is the first of its kind in this country, which hopes to deliver a coordinated and smooth transition of care after the patient is discharged from the hospital following a stroke. In the future, the better-coordinated transition of care combining shared care approach between tertiary and primary healthcare services will enable tracking of the patients’ progress and outcomes once they return to the community. It is hoped that with the shared care approach protocol, information on the long-term outcome measures i.e. clinical and rehabilitation parameters, will be available to the specialist stroke care team via the National Neurology Registry.

Clinical trials have shown that coordinating and ensuring the continuity of care for these patients following discharge from a tertiary care center can be extended to the community by primary care providers [31–33] with similar clinical and economic outcomes [27, 34]. Hence, the rationale for developing the iCaPPS, so as to provide better post stroke care delivery, is timely.

Variations in practice, which may occur during implementation of the iCaPPS, could be used as a feedback or an audit exercise on the process of care. Reviewing the workflow processes and making appropriate changes could also help overcome this issue, and it may further result in the improved quality of service delivery for the health center. Providing feedback on the weaknesses of the current workflow processes fosters focused remedial measures that can be taken to improve service delivery.

### Limitations

This was a first attempt to develop a care pathway for post stroke patients, using community-based resources in a healthcare system, which did not address collaborative multidisciplinary care once a patient is discharged from the hospital. Due to the heterogeneity of healthcare service provision throughout different parts of the country, with regards to post stroke care, identifying the relevant expert panel members from a small sampling was extremely challenging.

The effectiveness and impact on patients, as well as on staff workload, brought on by implementing the iCaPPS for the monitoring of post stroke patients is unknown. Technical expertise when using certain screening tools in public health centers that lack skilled staff members may prove to be a challenge. Training dedicated staff members to perform some of the assessments may be the only option if a shortage of expert staff members is an encountered problem in these public health centers.

This study only addressed the stroke care providers’ perspective on delivery of post stroke care after discharge from tertiary care. However, the input from stroke patients’ and their caregivers must also be considered to determine if the pathway addresses their needs, as this would be helpful to ensure the success of the pathway delivery.

### Recommendations

The iCaPPS will have to be evaluated in clinical trials to assess its effectiveness when compared with current practices. The cost effectiveness of using the iCaPPS over current practices will substantiate the guide’s feasibility when implementing the iCaPPS nationwide. Future research should also include evaluation on satisfaction of the service provided with the iCaPPS, by the patients and the caregivers.

## Conclusion

The iCaPPS is a useful guide through which to provide coordinated and comprehensive post stroke care delivery for patients who have been discharged to the community after an acute stroke, and among patients who are solely managed in primary care/community public health care centers after they present during later stages of stroke recovery. A study to test the effectiveness of the iCaPPS in terms of its feasibility to be used within the current healthcare delivery system, and its cost effectiveness should be explored.

## Abbreviations

FMS: Family Medicine Specialist
FMSA: Family Medicine Specialists’ Association (Malaysia)
iCaPPS: Integrated Care Pathway for Post Stroke
iCaPPS-Rehab©: Integrated Care Pathway For Post Stroke Patients-Rehabilitation Component
iCaPPS-Swallow©: Integrated Care Pathway For Post Stroke Patients-Swallowing Screen Component
